# Primary Care Provider Perceptions of Colorectal Cancer Screening Barriers: Implications for Designing Quality Improvement Interventions

**DOI:** 10.1155/2017/1619747

**Published:** 2017-01-10

**Authors:** Jennifer M. Weiss, Perry J. Pickhardt, Jessica R. Schumacher, Aaron Potvien, David H. Kim, Patrick R. Pfau, Elizabeth A. Jacobs, Maureen A. Smith

**Affiliations:** ^1^Division of Gastroenterology and Hepatology, University of Wisconsin School of Medicine and Public Health, Madison, WI, USA; ^2^Department of Medicine, University of Wisconsin School of Medicine and Public Health, Madison, WI, USA; ^3^University of Wisconsin Carbone Cancer Center, Madison, WI, USA; ^4^Department of Radiology, University of Wisconsin School of Medicine and Public Health, Madison, WI, USA; ^5^Department of Surgery, University of Wisconsin School of Medicine and Public Health, Madison, WI, USA; ^6^Department of Population Health Sciences, University of Wisconsin School of Medicine and Public Health, Madison, WI, USA; ^7^Department of Family Medicine, University of Wisconsin School of Medicine and Public Health, Madison, WI, USA

## Abstract

*Aims*. Colorectal cancer (CRC) screening is underutilized. Increasing CRC screening rates requires interventions targeting multiple barriers at each level of the healthcare organization (patient, provider, and system). We examined groups of primary care providers (PCPs) based on perceptions of screening barriers and the relationship to CRC screening rates to inform approaches for conducting barrier assessments prior to designing and implementing quality improvement interventions.* Methods*. We conducted a retrospective cohort study linking EHR and survey data. PCPs with complete survey responses for questions addressing CRC screening barriers were included (*N* = 166 PCPs; 39,430 patients eligible for CRC screening). Cluster analysis identified groups of PCPs. Multivariate logistic regression estimated odds ratios and 95% confidence intervals for predictors of membership in one of the PCP groups.* Results*. We found two distinct groups: (1) PCPs identifying multiple barriers to CRC screening at patient, provider, and system levels (*N* = 75) and (2) PCPs identifying no major barriers to screening (*N* = 91). PCPs in the top half of CRC screening performance were more likely to identify multiple barriers than the bottom performers (OR, 4.14; 95% CI, 2.43–7.08).* Conclusions*. High-performing PCPs can more effectively identify CRC screening barriers. Targeting high-performers when conducting a barrier assessment is a novel approach to assist in designing quality improvement interventions for CRC screening.

## 1. Introduction

Despite recent improvements in colorectal cancer (CRC) incidence and mortality, CRC remains the second leading cause of cancer-related death for men and women in the United States [1]. If screening modalities were optimally employed, CRC mortality would be largely preventable [2–9]. Although CRC screening is strongly endorsed by multiple professional societies and achievable using a variety of methods, national rates remain suboptimal, with only two-thirds of eligible individuals undergoing screening [10–12].

Previous efforts to improve CRC screening rates in the United States yielded an overall increase from ~50% to 65% over the past decade. However, rates have reached a plateau [13]. Evidence suggests targeting interventions to specifically identified barriers is likely to change practice [14]. This is critical to achieving further improvements in CRC screening rates, given the number and complexity of potential CRC screening barriers. However, as stated in a Cochrane review, “we do not yet know the most effective ways to identify barriers, to pick out from amongst all the barriers those that are most important to address, or how to select interventions likely to overcome them” [14].

Primary care providers (PCPs) are a logical source of information about barriers to CRC screening due to their integral role in cancer prevention. PCP recommendation is one of the strongest predictors of screening utilization, even with insurance disparities [15–19]. Yet it is not known which PCPs are best positioned to identify key CRC screening barriers in a health system. It is possible that targeting specific subgroups of PCPs (e.g., low- or high-performers) might yield the most comprehensive barrier assessment. In this study, we examine PCPs' identification of major barriers to CRC screening at each level of the healthcare system, determine if distinct groups of PCPs exist, and examine predictors of membership in these groups. Our results provide valuable information for determining which PCPs can reliably identify CRC screening barriers, with important implications for conducting barrier assessments prior to designing quality improvement interventions.

## 2. Materials and Methods

### 2.1. Study Design

The study was conducted in one of the 12 largest multispecialty physician groups in the United States. This group has approximately 1.7 million ambulatory visits per year, delivered by over 300 PCPs in more than 40 multispecialty and community-based primary care clinic sites.

A survey of CRC screening beliefs and practices was mailed to all PCPs within the physician group in February 2010. The goal was to gather data on the current landscape of CRC screening practices in the participating healthcare system prior to designing and implementing system-wide quality improvement interventions. The survey was based on the National Cancer Institute Survey of Colorectal Cancer Screening Practices, developed in collaboration with the CDC and Centers for Medicare and Medicaid Services, and has been used extensively in prior research [15, 20–22]. Survey items were divided into four sections: (1) cancer screening beliefs and practices; (2) attitudes toward CRC screening; (3) CRC screening modalities; and (4) provider characteristics. The overall survey response rate was 70% (*N* = 226/322). We present an analysis of a specific subset of survey items that assessed the importance of perceived barriers to CRC screening at the patient, provider, and system levels and restricted the sample to respondents who completed the entire subset of interest (*N* = 166/226), 73% of the survey responders. Survey responses were linked to patient panel, provider, and clinic level characteristics obtained from the electronic health record (EHR). This study was approved by the Institutional Review Board at the University of Wisconsin-Madison.

### 2.2. Survey Variables

Survey items assessed PCP perceptions of barriers to CRC screening at patient, provider, and system levels. Responses were dichotomized as “major barrier” or “minor/not a barrier.”* Patient-level barriers* included fear of finding cancer, belief that screening is not effective, embarrassment or anxiety about screening tests, lack of awareness of screening or perception of CRC as a nonserious health threat, fear of an invasive test, and concern about tolerating colonoscopy bowel prep.* Provider-level barriers* included the perception that PCPs do not routinely recommend screening to their patients and lack of time in clinic to discuss screening.* System-level barriers* included long wait times between ordering and scheduling a test, financial cost to patients, shortage of trained providers to conduct screening, and lack of a system for identifying patients eligible for screening. Two additional questions were included to assess provider and system-level barriers. The provider barrier question asked respondents if they had a method to identify which patients were in need of CRC screening exams. Answer choices were yes (coded as “minor/not a barrier”) or no (coded as “major barrier”). The system barrier question instructed respondents to “comment on the current capacity of facilities and personnel in your organization to meet the demand for performing colonoscopy.” Answer choices were “more than enough,” “just about right,” “inadequate,” and “don't know.” Responses were dichotomized with “inadequate” considered a “major barrier,” and “more than enough/just about right/don't know” coded as “minor/not a barrier.”

### 2.3. Patient, Provider, and Clinic Characteristics

Sample characteristics were obtained from the EHR. Patient variables included age, gender, race, marital status, primary language, insurance coverage, and comorbidities. A healthcare resource utilization score was calculated for each patient using Ambulatory Care Groups (ACG) based on outpatient and inpatient diagnoses from 12 months prior to survey administration [23, 24]. Provider variables included gender, specialty (Internal Medicine/Family Medicine), years in practice, CRC screening rates in 2009, and size of patient panel eligible for CRC screening. Clinic variables included clinic management and number of providers within the clinic. Clinic distance to the nearest colonoscopy facility was calculated using geographical software. Patients were assigned to PCPs using the plurality provider algorithm described by Pham et al. [25]. PCPs were assigned to clinics by the clinic at which the provider billed the majority of their Evaluation & Management (E&M) visits in 2009.

### 2.4. Identification of Screen-Eligible Population

We used EHR data to identify the pool of patients eligible for CRC screening in 2009 based on Healthcare Effectiveness Data and Information Set (HEDIS) metrics [26]. Adults aged 50–75 years were included if they were “currently managed” by the physician group. The definition of “currently managed” has been previously published [20]. Patients were excluded if they had a total colectomy based on ICD-9 codes and CPT codes.

### 2.5. Identification of CRC Screening Completion

Completion of CRC screening was defined as (a) fecal occult blood test (FOBT) in the prior 12 months, (b) flexible sigmoidoscopy, double contrast barium enema, or CT colonography in the past 5 years [11], or (c) colonoscopy in the past 10 years determined by HEDIS codes [26]. All PCPs in the physician group have access to colonoscopy and CT colonography, and most local third party payers cover CT colonography as a CRC screening option at the participating institution [27].

### 2.6. Statistical Analysis

Our primary goal was to determine if distinct categories of PCPs exist based on perceived CRC screening barriers. Cluster analysis was used to organize PCPs into meaningful structures based on survey responses [28]. Final identification of cluster groups was based on hierarchical cluster analysis using average linkage. Hierarchical clustering allows smaller clusters to be nested within larger ones reflecting a gradation of survey responses; average linkage allows clusters to be hierarchically related without depending on prior knowledge that the clusters resemble chains (single linkage) or are spatially compact (complete linkage) [29].

We compared the frequency of patient, provider, and clinic variables for the PCP cluster groups using *χ*^2^ tests for categorical variables and two-way analysis of variance tests for continuous variables. Multivariate logistic regression with robust estimation of standard errors and clustering at the clinic level was performed using the logit procedure in Stata to obtain odds ratios and 95% confidence intervals for provider and clinic level predictors of membership in one of the PCP groups. Analyses were conducted with Stata 12.0 (StataCorp, College Station, TX) and SAS 9.3 (SAS Institute, Cary, NC) software. All tests of significance used two-sided *p* values at the *p* < 0.05 level.

## 3. Results

### 3.1. Distribution of Survey Responses

**Figure 1:**
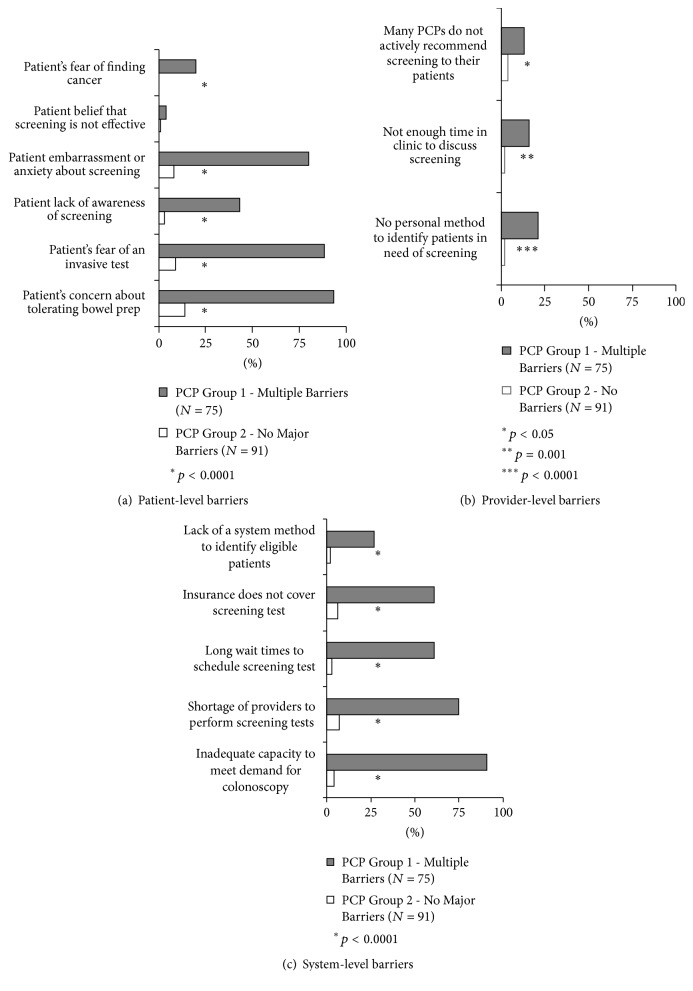
Distribution of survey responses across the two groups of providers for (a) perceived patient-level barriers, (b) perceived provider-level barriers, and (c) perceived system-level barriers.

Cluster analysis identified two groups of PCPs based on perceived barriers to CRC screening at patient, provider, and system levels: (1) PCPs who perceived multiple major barriers to CRC screening (*N* = 75) and (2) PCPs who perceived no major barriers to CRC screening (*N* = 91).  Figure 1 shows the percent of PCPs in each group who perceived each patient, provider, and system-level barrier as a major barrier.

### 3.2. Sample Characteristics

Overall, the 166 PCPs included in this analysis worked at 24 primary care clinics and cared for 39,430 patients eligible for CRC screening, according to the methodology previously described. The majority of patients were 50–60 years old, White, married, primarily English speaking, and covered by commercial insurance (Table 1). Of the 166 PCPs, 52% were female, over half practiced Internal Medicine (52%), and two-thirds had practiced >10 years. The average number of patients eligible for CRC screening in a provider's panel was 238. Two-thirds of the primary care clinics were physician-owned with an average distance of 7.6 miles to the nearest colonoscopy facility.

PCPs who perceived multiple barriers to CRC screening (*N* = 75) cared for 26,420 patients eligible for screening and worked at 14 different primary care clinics. Comparatively, PCPs who perceived no major barriers to CRC screening (*N* = 91) cared for 13,010 patients eligible for screening and worked at 10 different primary care clinics. The patients assigned to PCPs who perceived multiple barriers were more often female (61% versus 48%, *p* < 0.001), spoke English as a primary language (92% versus 89%, *p* < 0.001), and had more commercial insurance coverage (67% versus 63%, *p* < 0.001). There was no significant difference between the percent of patients with congestive heart failure and diabetes mellitus or the average ACG resource utilization score between the patients assigned to the two groups. Providers who perceived multiple barriers were more often female (61% versus 45%, *p* = 0.043), had a higher CRC screening rate on average (66% versus 59%, *p* = 0.013), and on average had a larger patient panel eligible for CRC screening compared to the PCPs who perceived no major barriers (250 versus 91 patients, *p* < 0.001). There was no significant difference in clinic characteristics between the two groups of PCPs.

### 3.3. Predictors of Membership in PCP Groups

In the adjusted model with provider and clinic characteristics, the most significant predictor of membership in one of the PCP groups was provider CRC screening rate (Table 2). PCPs identifying multiple barriers were more likely to be in the top half in terms of CRC screening performance (OR, 4.14; 95% CI, 2.43–7.08). The mean CRC screening rate for the top performers compared to the bottom performers was 75% versus 49% (data not shown). PCP gender, specialty, and years in practice were not significant predictors of membership in a PCP group.

## 4. Discussion

Our study identified two distinct groups of primary care providers for assistance with CRC screening barrier assessment: (1) PCPs who perceive multiple major barriers to CRC screening and (2) PCPs who perceive no major barriers. The PCPs identifying multiple barriers were four times as likely to be in the top half in terms of CRC screening performance compared to PCPs identifying no major barriers. We hypothesize that high-performers were able to identify barriers at multiple levels of the healthcare system because they are more actively engaged in the CRC screening process and therefore encounter and are attuned to more barriers. This is a critical finding, as the identification of key barriers to screening may allow for the development of targeted interventions to improve CRC screening at a time when rates have plateaued [13].

We found that the PCPs who identified more barriers to CRC screening accurately assessed the climate of our healthcare system at the time of the survey. At the time of survey administration there was a shortage of gastroenterologists in our healthcare system to perform colonoscopies, inadequate capacity to meet the demand for colonoscopies, and very long wait times (>1 year) between ordering and scheduling the exam. These PCPs also identified lack of a system for identifying patients eligible for screening as a major barrier. Although all clinics and PCPs used the same EHR, there was no standard alert at the time of the survey. In response to the survey data, more gastroenterologists were hired to address these issues, significant changes were made to the scheduling process, and the wait time was successfully decreased to <3 months. In addition, a system-wide health maintenance alert was developed for patients overdue for CRC screening and was implemented for all clinics and PCPs through the EHR.

Interestingly, few PCPs in both groups felt that PCPs not actively recommending screening to their patients was a major barrier. This is despite the fact that the large majority of patients who are not current with CRC screening, 94% of those are over 50 years old [16] and 84% of those over 65 years old [17], list no physician recommendation as a major reason. This may be due to the fact that PCPs are not aware of their degree of influence on a patient's decision to be screened or they think that the majority of PCPs are recommending CRC screening to all eligible patients. Also of interest is that PCP years in practice did not predict membership in a PCP group. This is contrary to some studies that suggest that providers who have been in practice longer may be less likely to deliver high-quality care, possibly due to out of date information or rapidly changing guidelines [30, 31].

There is significant evidence that interventions tailored to specific barriers can improve care delivery. In a large meta-analysis, Baker et al. [14] compared interventions designed to improve receipt of preventive care that were either tailored or not tailored to address identified barriers and compared these groups to a no-intervention control group. They concluded that interventions tailored to prospectively identified barriers are more likely to improve practice. Tailoring interventions to increase CRC screening is critical, due to the complexity of barriers that results from multiple available CRC screening modalities, the number of providers required for the process, and the various locations where screening can be performed.

Baker et al. [14] also noted that the methods used to identify barriers varied widely between the studies; therefore, the best methods for identifying barriers require further research. It is common practice to call upon high-performing providers and healthcare systems to share their “best practices” so that lower-performers can learn how to improve their CRC screening rates [32]. In addition, we often see low-performing providers interviewed or surveyed about the barriers that are preventing them from being a “high-performer.” Our results suggest that lower-performing providers are less likely to identify barriers at different levels within the healthcare system. The results also suggest that initial barrier assessments should target high-performing providers to identify pertinent barriers and possible facilitators to assist in the design and implementation of interventions to improve CRC screening rates.

There are limitations to this study. First, we report findings from a large academic physician group, which could impact generalizability. However, large multispecialty systems are quickly becoming a preferred way to provide high-quality health care and are therefore critical to the understanding of modern health care delivery [33]. Second, our nearly all-white patient population may limit potential generalizability to healthcare systems with a more diverse patient population. Third, the survey reports perceptions of barriers to CRC screening in our healthcare system in 2009-2010. While this was more than five years ago, there is no reason to believe that perceptions of barriers have fundamentally shifted since that time. In addition, our study shows that high-performers are able to more accurately identify CRC screening barriers within a healthcare system and this same concept can be applied to other systems where the exact details of the barriers may be different. Fourth, there are inherent selection biases when relying on survey data, such as nonresponse bias [34–36]. This impact is likely minimal due to our high response rate. Typical survey response rates for healthcare providers are well below 50% [37]. Our overall response rate was 70%; the sample for this study included providers with complete responses to the questions of interest which is 51% of the overall sample. Fifth, a number of our variables rely on the EHR, which could result in missing data and possible misclassification of a completed screening test. However, this is unlikely to result in a systematic bias across clinical settings since all clinics used a fully integrated EHR that has been populated with all data since 1991 (including scanned documents that were manually reviewed to assess completion of CRC screening outside the system). Finally, our cluster analysis used dichotomized survey responses (major barrier versus minor/not a barrier) which results in a loss of some of the finer details in the survey data. However, this was necessary due to the sample size.

## 5. Conclusions

Multiple healthcare organizations across the country have signed the American Cancer Society call for screening 80% of eligible patients for colorectal cancer by 2018. In order to reach this goal, large initiatives will be formed. In this study, we identified two groups of PCPs: (1) PCPs who perceive multiple major barriers to CRC screening and (2) PCPs who perceive no major barriers. PCPs identifying multiple barriers were more likely to be in the top half in terms of CRC screening performance, suggesting that high-performers of quality metrics of interest should be targeted as the source for effective barrier identification prior to the design and implementation of interventions. Future research will involve determining if the impact and sustainability of these interventions are enhanced for providers who identified the barriers or if the impact is the same for all PCPs.

## Figures and Tables

**Table 1:** Sample characteristics of patients, providers, and clinics (overall and by provider perceptions of barriers).

	Overall	Multiple perceived barriers to CRC screening	No perceived barriers to CRC screening	*p* value
*Patient characteristics*	*N* = 39,430	*N* = 26,420	*N* = 13,010	
Age, %				<0.001
50–54	27	27	27	
55–59	27	27	26	
60–64	21	21	21	
65–69	14	14	14	
70–75	11	11	12	
Gender, %				<0.001
Female	56	61	48	
Race, %				0.046
White	93	93	92	
Marital status, %				0.004
Married	71	70	72	
Language, %				<0.001
English (as primary language)	91	92	89	
Primarily non-English	1	1	1	
Unknown	8	7	10	
Insurance, %				<0.001
Commercial	66	67	63	
Medicare	24	24	25	
Medicaid or uninsured	2	2	2	
Missing	9	8	10	
Comorbidities, %				
Congestive heart failure	1	1	1	0.835
Diabetes mellitus	9	9	10	0.437
Hypertension	36	36	35	0.005
ACG resource utilization score (mean, SD)	0.58 (0.42)	0.58 (0.42)	0.58 (0.43)	0.537

*Primary care provider characteristics*	*N* = 166	*N* = 75	*N* = 91	
Age, %				0.470
30–39	24	20	27	
40–49	26	29	23	
50–59	25	28	22	
60–69	9	11	8	
Missing	16	12	20	
Gender, %				0.043
Female	52	61	45	
Specialty, %				0.133
Internal medicine	52	53	52	
Family medicine	48	47	48	
Years in practice, %				0.358
<10 yrs	18	15	21	
10–20 yrs	31	33	30	
>20 yrs	35	40	31	
Missing	16	12	18	
Average CRC screening rate (%) in the year prior to survey administration (mean, SD)	62 (17.5)	66 (15.7)	59 (18.4)	0.013
Number of patients eligible for CRC screening in a provider's panel (mean, SD)	238 (232)	250 (182)	91 (128)	<0.001

*Primary care clinic characteristics*	*N* = 24	*N* = 14	*N* = 10	
Clinic management, %				0.77
Physician-owned	67	64	70	
Hospital-owned	33	36	30	
Distance to nearest colonoscopy center in miles, (mean, SD)	7.6 (5.6)	7.1 (3.8)	8.5 (7.7)	0.564
Number of providers within the clinic, (mean, SD)	6.9 (4.9)	8.4 (5.5)	4.8 (3.0)	0.074

SD = standard deviation, ACG = ambulatory care group, and CRC = colorectal cancer.

**Table 2:** Adjusted odds ratios and 95% confidence intervals for provider and clinic predictors of membership in PCP group perceiving multiple barriers (*N* = 166).

	OR	95% CI	*p* value
*Primary care provider characteristics*			
Gender			
Male	(ref)		
Female	2.13	0.88, 5.13	0.090
Specialty			
Family medicine	(ref)		
Internal medicine	0.69	0.19, 2.37	0.552
Years in practice			
<10 yrs	(ref)		
10–20 yrs	1.12	0.53, 2.35	0.764
>20 yrs	1.24	0.52, 2.92	0.626
Missing	0.69	0.24, 2.00	0.498
CRC screening rate			
Bottom half	(ref)		
Top half	4.14	2.43, 7.08	<0.0001

*Primary care clinic characteristics*			
Clinic management			
Hospital-owned	(ref)		
Physician-owned	0.53	0.18, 1.57	0.253
Distance to nearest colonoscopy center in miles	0.97	0.87, 1.07	0.520
Number of providers within the clinic	0.95	0.90, 0.99	0.044

OR = odds ratio, CI = confidence interval, ACG = ambulatory care group, and CRC = colorectal cancer.
